# Unveiling the Immunomodulatory Potential of Phenolic Compounds in Food Allergies

**DOI:** 10.3390/nu16040551

**Published:** 2024-02-16

**Authors:** Rodolfo Simões, Ana Catarina Ribeiro, Ricardo Dias, Victor Freitas, Susana Soares, Rosa Pérez-Gregorio

**Affiliations:** 1 REQUIMTE/LAQV, Departamento de Química e Bioquímica, Faculdade de Ciências da Universidade do Porto, Rua Campo Alegre 687, s/n, 4169-007 Porto, Portugal; up202004095@edu.fc.up.pt (R.S.); catarinaribeiro23@gmail.com (A.C.R.); ricardo.dias@fc.up.pt (R.D.); vfreitas@fc.up.pt (V.F.); susana.soares@fc.up.pt (S.S.); 2 Food and Health Omics Group, Food and Agroecology Institute, University of Vigo, Campus As Lagoas, s/n, 32004 Ourense, Spain; 3 Food and Health Omics Group, Department of Chemistry and Biochemistry, Galicia Sur Health Research Institute (IISGS), SERGAS-UVIGO, 32002 Ourense, Spain

**Keywords:** food allergies, oral tolerance, phenolic compounds, digestion, immune system, human microbiota

## Abstract

Food allergies are becoming ever more prevalent around the world. This pathology is characterized by the breakdown of oral tolerance to ingested food allergens, resulting in allergic reactions in subsequent exposures. Due to the possible severity of the symptoms associated with this pathology, new approaches to prevent it and reduce associated symptoms are of utmost importance. In this framework, dietary phenolic compounds appear as a tool with a not fully explored potential. Some phenolic compounds have been pointed to with the ability to modulate food allergies and possibly reduce their symptoms. These compounds can modulate food allergies through many different mechanisms, such as altering the bioaccessibility and bioavailability of potentially immunogenic peptides, by modulating the human immune system and by modulating the composition of the human microbiome that resides in the oral cavity and the gastrointestinal tract. This review deepens the state-of-the-art of the modulation of these mechanisms by phenolic compounds. While this review shows clear evidence that dietary supplementation with foods rich in phenolic compounds might constitute a new approach to the management of food allergies, it also highlights the need for further research to delve into the mechanisms of action of these compounds and decipher systematic structure/activity relationships.

## 1. Introduction

Over the past few years, allergic disorders have become more common around the world, and it is estimated that adults (up to 3%) and children (up to 8%) in industrialised countries are affected by food allergies [1]. The severity of food allergy symptoms, along with the rising prevalence of these pathologies, places great importance on the development of novel therapeutic approaches [2,3,4,5]. The oral exposure to food allergens can result in three distinct categories of allergic responses: IgE-mediated responses, cell-mediated responses and mixed responses, dependent on both IgE and the immune cells [6]. Some of the most common food items capable of initiating allergic reactions include eggs, fish, soybeans, tree nuts, milk, peanuts, shellfish and wheat [7,8].

Typically, patients develop food allergies in their early childhood and the pathology is resolved by the time they reach adulthood. However, food allergies can persist into adolescence and adulthood. Factors such as the age of diagnosis, timing of resolution, nature of immune responses and associated comorbid allergic diseases determine the severity of this pathology [3,9,10].

There are a multitude of diagnostic methods available, with skin prick tests and food-specific IgE serum testing being among the most used. Patient’s medical history and the nature and severity of food allergies should be considered when selecting an appropriate diagnostic technique. Avoiding food allergens through dietary elimination combined with being prepared to quickly address allergic reaction using epinephrine are the prevailing approaches to manage food allergies and prevent anaphylactic reactions. However, these approaches are not ideal, since they do not address the alterations in cellular mechanisms that lead to food allergies. Furthermore, nutritional deficits may ensue from the exclusion of particular foods [11].

IgE-mediated allergic reactions initiate rapidly after the ingestion of allergic foods, with some of the symptoms being shortness of breath, wheezing, coughing, nausea, vomiting and in extreme cases, anaphylactic reactions [12].

Recently, the use of phenolic compounds in the prevention and mitigation of symptoms of these pathologies has been proposed. Both the scientific community and the general population have asserted that the consumption of plant-based foods is good for overall human health, due to their anti-aging, anti-inflammatory and anti-microbial properties [13,14,15,16]. This has been in part attributed to the high concentration of phenolic compounds present in these foods. In addition, foods rich in phenolic compounds have shown promise in the modulation of food allergies, allowing for the development of new dietary approaches to regulate food allergies.

This review will thus focus on the various mechanisms through which phenolic compounds can modulate IgE-mediated food allergies, namely altering the digestion of allergenic proteins and the modulation of the phenotype and function of immune system cells and their interactions with the commensal microbiota that exists through the gastrointestinal tract [3,17]. This review will also address the current research regarding each of these topics, as always identifying the existing gaps in knowledge.

## 2. Biologic Systems Influencing the Development of Food Allergies

The maintenance or loss of oral tolerance is dependent on the interaction between a myriad of biological systems, with emphasis on the human gastrointestinal tract. As such, phenolic compounds have the ability to modulate food allergies at various levels, with some of the most prominent being the digestion of allergenic proteins, the intestinal absorption of potentially immunogenic peptides, the phenotype and function of immune cells and the human commensal microbiomes that exist in the mouth and in the gut [18,19,20].

### 2.1. Digestion of Food Allergens and Its Influence of Food Allergies

Orally ingested antigens start to be digested as soon as they enter the oral cavity, both by salivary enzymes (mainly α-amylase) and by mechanical maceration during mastication [21]. Both cleave the 1,4-glycosidic bonds of carbohydrates due to the action of α-amylase and the breakdown of foods facilitates the action of gastric and intestinal enzymes, further aiding in the digestion of foods. Ingested proteins are then further digested in the stomach, where the acidic pH both denatures proteins and activates the gastric enzyme pepsin [22,23]. Finally, proteins enter the intestine, where the release of pancreatic enzymes like chymotrypsin and trypsin further degrade proteins into peptides of varying lengths, which might then be sampled and presented to the cells of the immune system, initiating allergic reactions [24].

Protein stability can be classified as the ability of proteins to preserve their native conformation and structure after physical and chemical digestion [25]. The stability of different proteins can vary greatly, and it is influenced by a myriad of factors, both intrinsic and extrinsic to proteins [26]. To start, the digestion of proteins is itself dependent on many factors. The pH of gastric fluids can vary greatly, ranging from two during fasting to as high as six during the ingestion of certain foods. The optimal pH of pepsin is between 1.8 and 3.2, and as such the activity of this enzyme also fluctuates with the pH of the stomach [27]. The composition of food matrices also plays an important role in the digestion of proteins. Meals with a high fat content have an increased transit time throughout the gastrointestinal tract, thus exposing them to the effect of digestive enzymes for longer. The stability of proteins is thus also linked to the action of lingual, gastric and pancreatic lipases that play a role in the digestion of food matrixes [28].

Regarding the intrinsic characteristics of proteins, the main factors determining their stability are their structure and three-dimensional conformation. Proteins with a stable three-dimensional structure due to, for example, the presence of disulphide bonds, might be able to resist degradation in the harsh conditions of the gastrointestinal tract, remaining virtually intact [29].

All of these factors contribute to the great heterogeneity of protein digestion, with some proteins being extensively broken down into smaller peptides, while others pass through the gastrointestinal tract without being significantly degraded [30]. In the past, protein stability has been proposed as a measure of the immunogenic potential of different proteins, seeing as more stable proteins could preserve their conformational epitopes and elicit allergic responses. However, authors have recently reported the ability of different immunogenic peptides to elicit the same types of allergic responses. This occurs due to the preservation of epitopes in these peptides that maintain the ability to bind to human IgE and initiate allergic responses. Due to this, protein stability alone is not an accurate predictor of immunogenic potential and must be coupled with other tests, such as IgE binding assays, to access the potential of different protein sources to initiate allergic reactions [31,32].

### 2.2. Bioavailability of Food Allergens and Its Effect on Food Allergies

Allergens can cross the intestinal barrier through many different pathways. One common pathway is the paracellular pathway, through which small molecules can cross the intestinal barrier though tight junctions [33,34]. Tight junctions are intercellular multi-protein complexes composed mainly of four types of proteins: claudins, occludins, junctional adhesion molecules (JAMs) and tricellulin [35]. The intestinal uptake of different molecules through these molecular structures is determined by their size and charge [33]. However, the selectivity of the paracellular pathway can decrease drastically after disruptions of the intestinal barrier, such as the inflammation of intestinal tissues and differential expression of the proteins that compose tight junctions [36]. TNF-α and IFN-γ were reported to alter the expression of tight junction proteins, increasing the permeability of the intestinal barrier and resulting in a loss of selectivity [37].

Molecules can also traverse the intestinal barrier via the transcellular pathway. The epithelial cells of the intestinal barrier possess transporters such as sodium-dependent transporters of glutamine, glucose and alanine. These transporters translocate small molecules across the intestinal barrier. Larger molecules, such as allergenic peptides and proteins, can also be incorporated into vesicles to cross the intestinal barrier [38]. Luminal antigens are sampled into the gut-associated lymphoid tissues (GALTs) through M cells, goblet cells and CX3CR1 macrophages [6]. The antigens sampled by these cells are then presented to immune system cells, thus establishing an oral tolerance to orally ingested allergens [6].

Authors have also reported changes in intestinal function after the breakdown of oral tolerance to food antigens. Altered carbohydrate absorption patterns were reported in food-allergic patients after allergen challenge, while healthy controls and allergic patients under allergen exclusion diets presented normal absorption patterns [39]. These changes can be attributed to the changes in the function of the intestinal barrier after gut inflammation, as the normal function of cells is altered, and the sampling process of dietary antigens is affected. A leaky gut may reduce the selectivity of tight junctions, increasing the influx of potentially allergenic proteins and peptides [40]. The altered function of cells can also result in differences in the sampling process, thus promoting an allergic state and potentiating allergic reactions upon re-exposure to the same allergen [19].

### 2.3. The Human Immune System and Its Role in Food Allergies

The human immune system also plays an important role in IgE-mediated food allergies. Antigens sampled from the gastrointestinal tract are transported to draining lymph nodes and are presented to T cells by DCs [41]. In the presence of retinoic acid and TGF-β, this presentation of antigens promotes the differentiation of naive T cells into T regulatory cells (Tregs) [42]. After differentiation, the integrin α4β7 expressed by Tregs cells aids their translocation to the lamina propria, where these cells maintain tolerance to food allergens through the secretion of cytokines such as TGF-β and IL-10. These cytokines suppress mast cells and also promote the maintenance of IgA in the lumen [43].

Food allergies are characterised by alterations in these cellular mechanisms. Sensitization occurs upon exposure to food allergens, with subsequent exposures to the identical antigen resulting in an inflammatory response [5].

The release of IL-25, IL-33 and TSL promotes DC activation and the differentiation of type 2 innate lymphoid cells (ILC2s) [44]. Activated DCs have an upregulation of the expression of the surface marker OX40L. After transporting food allergens to the draining lymph nodes, the OX40L present on the surface of activated DCs binds to OX40 on the surface of naive T cells upon antigen presentation, promoting the differentiation of these cells into Th2 cells [45]. An increase in the Th2/Th1 cell ratios is characteristic of this pathology, as Th2 cells are associated with pro-inflammatory responses [46]. Proinflammatory cytokines (e.g., IL-5 and IL-13) are then secreted by these cells, promoting basophil and eosinophil recruitment to gut tissues, further promoting allergic sensitization. The pro-inflammatory cytokines also promote the expression of inflammatory markers by epithelial cells, resulting in a polarisation of intestinal macrophages into an alternatively activated M2 phenotype [47]. Finally, the IL-4 secreted by these cells promotes B cells class switching, inducing the production of dietary-specific IgE [48]. It has also been documented that naive T cells can differentiate into T helper 9 cells. Through the secretion of IL-9, these cells facilitate the accumulation of mast cells that reside in tissues, thereby stimulating the accumulation of mast cells that reside in tissues and contributing further to the development of allergic responses [49].

**Figure 1:**
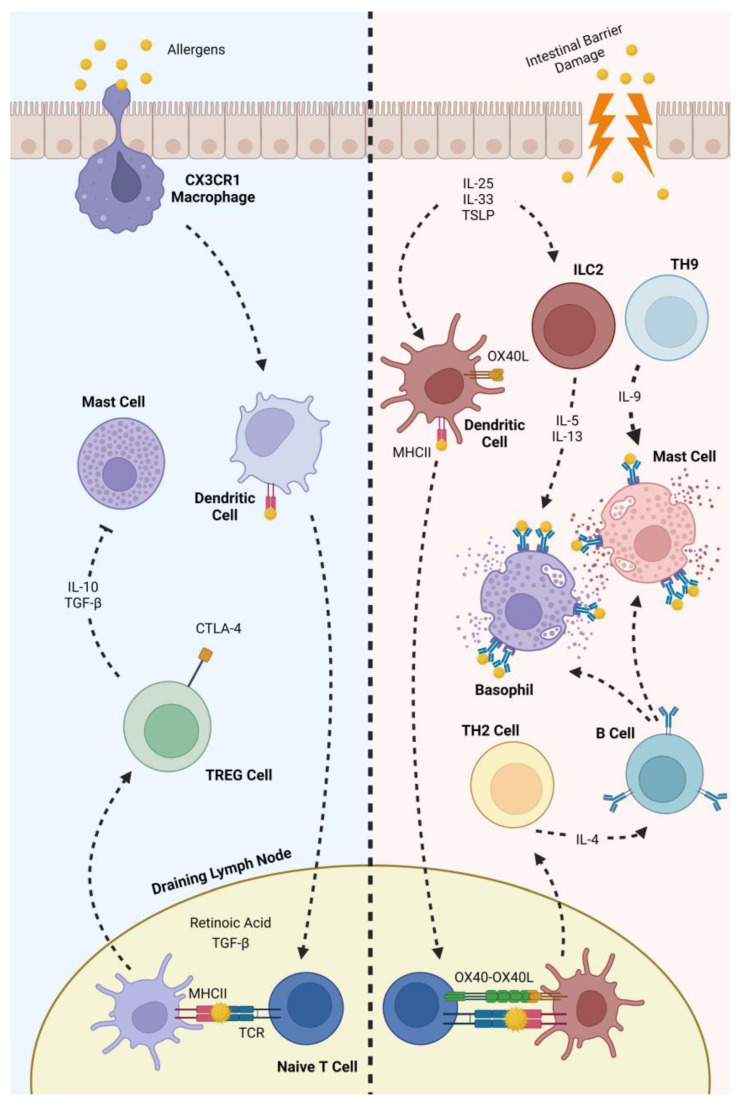
Left—maintanence of oral tolerance; right—allergic response to food allergens.

After this, subsequent exposures to the same allergen result in allergic reactions. Allergen-specific IgEs recognize the epitopes and bind to them, then cross-link with the receptor FcεRI present in the surface of mast cells and basophils, resulting in the downstream activation of the MAPK and NFκB signalling pathways. This results, among other things, in the degranulation of these cells and the release of pro-inflammatory mediators such as histamine, serotonin, proteases and proteoglycans [50]. All of the previous cellular events are summarized in Figure 1.

### 2.4. The Human Commensal Microbiota and Its Role in Food Allergies

Another key factor in the modulation of food allergies is the human commensal microbiome that resides in the oral cavity and all through the gastrointestinal tract [51]. The genetic signatures and metabolites produced by these microorganisms possess immunomodulatory properties, influencing the normal development of many organs and altering the host’s immune response and metabolism [52,53,54,55].

More than a thousand species of microorganisms colonise the human oral cavity and gastrointestinal tract. The formation and maintenance of the host’s immune system, the digestion processes of foods, epithelial cell differentiation, and many other physiological processes are all significantly influenced by these commensal bacteria [56,57]. Throughout the gastrointestinal tract, commensal bacteria are typically located in mucosal layers. They can also form biofilms on the surface of hard tissues, for example, teeth. Antimicrobial peptides, IgAs and mucins act as chemical barriers that prevent the proliferation of pathogenic microbes and reduce the risk of infection [58,59].

The human commensal microbiome composition is not static, being modulated by genetics, hygiene practices, disease status, drug intake and of course, diet patterns. The major phyla present in the oral microbiome are *Proteobacteria* (genus Haemophilus, Neisseria), *Firmicutes* (genus Haemophilus, Gemella, Veillonella and Granulicatella), *Bacteroidetes* (mainly represented by Prevotella), *Actinobacteria* (genus Corynebacterium, Actinomyces, Rothia) and *Fusobacteria* (genus Fusobacterium). The gut microbiome is composed primarily by four major microbial phyla: *Bacteroidetes*, *Firmicutes*, *Actinobacteria* and *Proteobacteria*. As previously mentioned, the composition of the microbiota is not static, being determined by factors such as age, diet and lifestyle. In early life, the gut microbiome is composed primarily of *Proteobacteria*. The composition of the gut microbiome is then dominated mainly by *Actinobacteria*, before finally maturing into the microbiota characteristics of adult individuals, dominated by *Firmicutes* and *Bacteroidotes*. A great variety of techniques can be used to study the human microbiome, with the 16S rRNA gene analysis using a clone library being one of the most common [60].

The role of human commensal microbiome is fundamental in many human biological processes, such as controlling epithelial development, modifying metabolic phenotype, and regulating innate immunity [61,62,63]. Different diseases have been linked with changes of the human intestinal microbiome, such as diabetes mellitus, cirrhosis, metabolic syndrome, inflammatory bowel disease, atherosclerosis, obesity, hepatocellular carcinoma and alcoholic liver disease [58,64].

Microbe-associated molecular patterns (MAMPs) present on the membrane of the bacteria that compose the human commensal microbiome bind with pattern recognition receptors present in the membrane of immune system cells, priming these cells to eliminate pathogens, while also promoting tolerance. Upon the recognition of MAMPs, cytokines that influence immune responses are released by the immune and epithelial cells. Some of the innate immune cell types are also crucial for the maintenance of oral tolerance, including dendritic cells, basophils, mast cells, macrophages and ILC. The adaptive immune response depends on B and T cells and, together with the innate system, is a crucial component of allergic responses [65].

Commensal bacteria can also influence immune responses by interacting with the cells of the adaptive immune system, namely T-cells. Some commensal intestinal bacteria promote the secretion of IL-1β in the intestinal lamina propria. This in turn leads to an increase in the population of T helper type 17 (Th17) cells [65]. Bacterial antigens, such as polysaccharide A, can also promote oral tolerance in hosts by promoting increases in Treg cells populations and promoting IL-10 secretion [66].

Finally, the commensal bacteria’s metabolism generates numerous molecules capable of modulating the oral tolerance to food allergens. A bioproduct of dietary fibre fermentation in the gastrointestinal tract, short-chain fatty acids (SCFAs), such as pentone, acetate and propionate, are one of these secondary metabolites. These metabolites are capable of modulating the human immune system, contributing to the maintenance of the oral tolerance to food allergens [67,68]. SCFAs serve as diffusible signalling molecules, binding to the G protein-coupled receptors (GPRs) expressed by cells such as epithelial cells and immune cells, initiating various signalling cascades involved in the maintenance of the oral tolerance to food allergens [69]. SCFAs are also strong lysine deacetylase and histone deacetylase inhibitors, with the ability to modulate the proliferation and differentiation of immune system cells by altering the expression of genes. Increases in histone acetylation via the inhibition of lysine deacetylase and histone deacetylase also decrease the release of proinflammatory cytokines (such as IL-6, IL-8 and TNF-α) and increase intestinal Treg levels and IgA production [3].

Due to the crucial role of these microorganisms in many biological processes, changes in the microbial populations present in the oral cavity and the gastrointestinal tract are characteristic of many pathologies.

Dysbiosis of the host’s microbiota is also characteristic of food allergy [70,71,72]. As mentioned before, many different factors can alter the compositions of these populations of commensal bacteria, with one of the most influential being the diet [51]. Dietary fibres display a prebiotic effect, promoting the growth of particular bacteria and profoundly influencing the fermentative metabolism of the commensal bacterial microbiome [73]. Conversely, dysbiosis is exacerbated by high-fat diets, which are linked to immune system dysregulation and possible oral tolerance loss [74,75].

## 3. Phenolic Compounds and Their Bioactivity

Dietary polyphenols found in many foods have emerged as candidates for the development of new therapeutic approaches to both prevent food allergies and reduce associated symptoms. There are more than 8000 phenolic compounds synthesized by plants, presenting a great structural variety. Phenolic compounds are synthesized by the secondary metabolism of plants and therefore, occur in all plant-based food and derived products [76].

Many authors have extensively described both the in vitro and in vivo bioactivity of phenolic compounds, namely their anti-inflammatory and antioxidant properties [77]. Many studies have also been proposed to study the use of dietary phenolic compounds as modulators of allergic reactions (Table 1).

**Figure 2:**
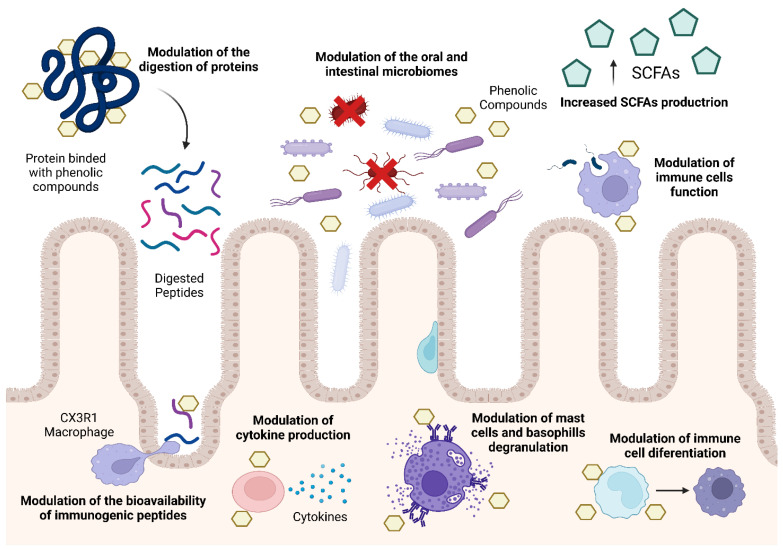
Main mechanisms of modulation of food allergies by phenolic compounds.

The following sections of this review will explore the chemistry and bioactivity of polyphenols, as well as the three main mechanisms of modulation by phenolic compounds (Figure 2): the modulation of the bioaccessibility and bioavailability of potentially immunogenic peptides, the modulation of the human immune system, and finally, the modulation of the commensal bacteria that reside in the oral cavity and the gastrointestinal tract.

### 3.1. Chemistry of Phenolic Compounds

To further understand the mechanism through which phenolic compounds can modulate food allergies, it is important to understand the general structure and chemical composition of this class of plant metabolites. All phenolic compounds share a common structural feature containing at least one phenol ring with one or more hydroxyl groups in their structure. This class of chemical compounds possesses a great variety in structure, which allows for a wide range of biological activities [84,85].

**Figure 3:**
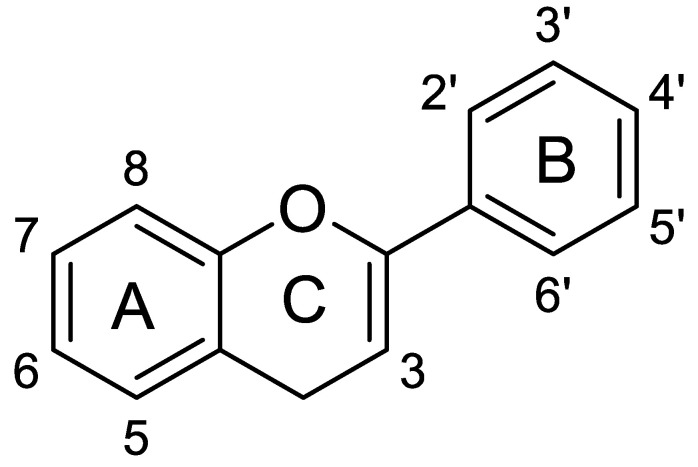
Basic structure of flavonoids.

Phenolic compounds are classified into two groups: non-flavonoids and flavonoids. Flavonoids constitute approximately two-thirds of the phenolic compounds found in foods. The basic structure of this subclass of phenolic compounds is composed of two phenolic rings (A and B), connected to a heterocyclic pyran ring (C) (Figure 3).

**Figure 4:**
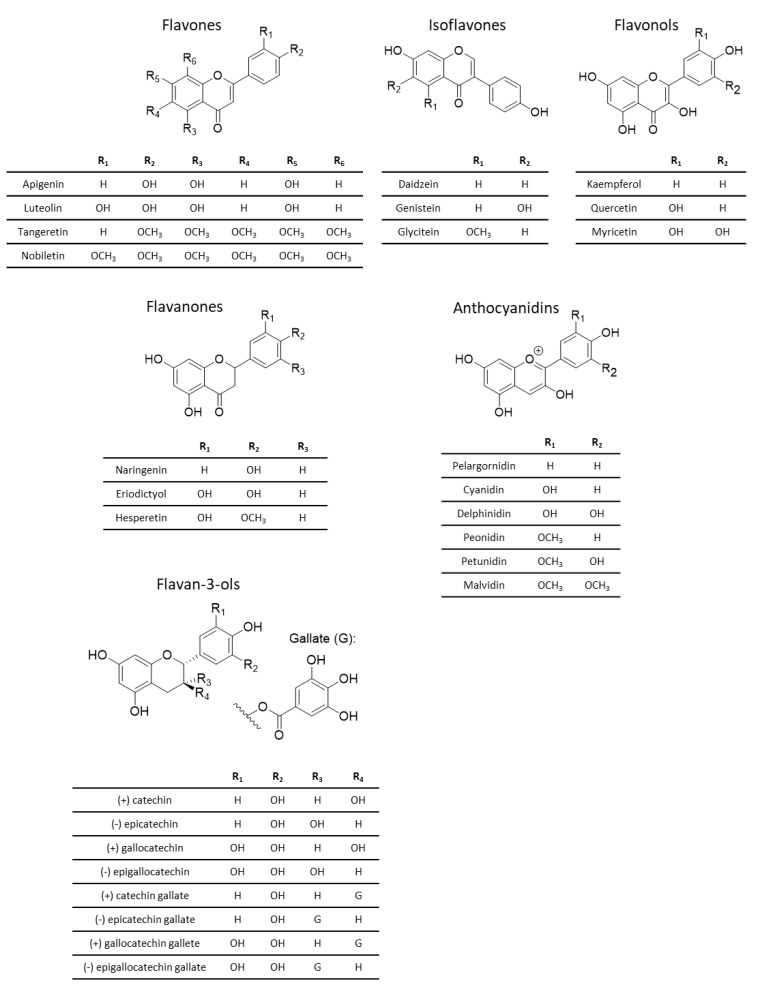
Basic structure of the six subclasses of flavonoids.

Flavonoids are further divided into six subclasses, depending on the structure of their heterocyclic ring. These six subclasses are flavan-3-ols, anthocyanins, flavanones, flavones, flavonols and isoflavones (Figure 4) [84,86].

**Figure 5:**
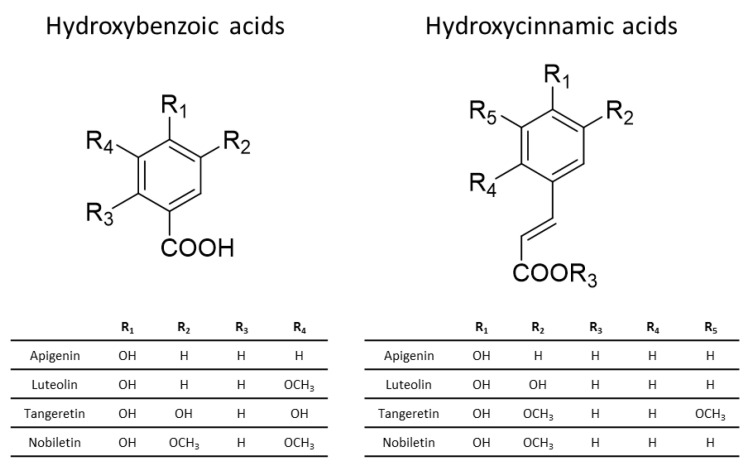
Basic structure of hydroxycinnamic and hydroxybenzoic acids.

Non-flavonoid phenolic compounds are those that lack the fundamental structure found in flavonoids. Although the chemical structures of these compounds differ significantly, the majority of them are smaller in size and more basic in nature compared to flavonoids. Non-flavonoids are classified into numerous subclasses, with the most prevalent being phenolic acids, the most abundant form of dietary phenolic compounds. Phenolic acids are further classified as hydroxybenzoic acid-derived phenolic acids or hydroxycinnamic acid-derived phenolic acids (Figure 5) [84,87].

### 3.2. Metabolization of Phenolic Compounds

When orally ingested, phenolic compounds are extensively metabolized. Most of the phenolic compounds in plant tissues occur in the form of glycosides and esters [88,89], but the ingested compounds are degraded as they pass through the gastrointestinal tract. This metabolization can significantly alter not only their chemical structure but also their bioactivity. As such, their immunomodulatory properties might also be affected by this.

**Figure 6:**
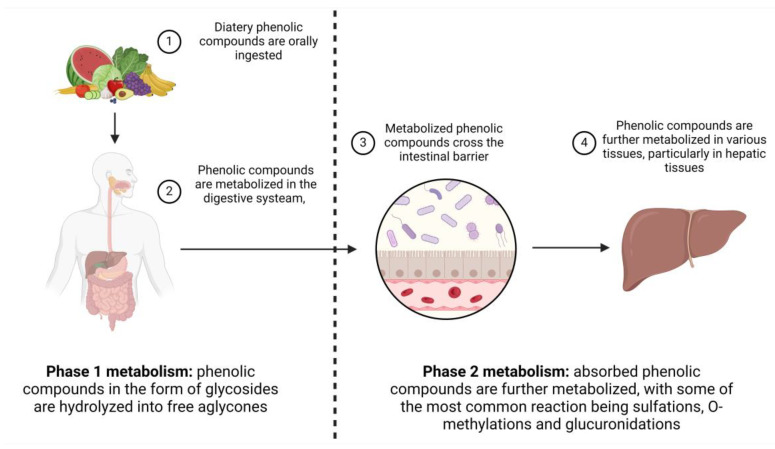
Schematic representation of the metabolic pathway of phenolic compounds in humans. Left—Phase 1 metabolisms (1 and 2); Right—Phase 2 Metabolism (3 and 4).

The process of metabolization can be divided into two stages: the initial stage comprises reduction, oxidation and hydrolysis and the second stage comprises glucuronidation and methylation. The metabolization of phenolic compounds begins immediately following the oral ingestion of phenolic compounds and continues even after their absorption in the gastrointestinal tract. Certain compounds undergo extensive metabolization, not only in the gastrointestinal tract but also in different body tissues. Conversely, phenolic compounds with lower molecular weights and simpler structures are absorbed in the large intestine with minimal alterations to their chemical structure (Figure 6). The gut microbiome also plays a crucial role in phenolic compound metabolism. Substances that are not absorbed in the small intestine undergo metabolism by the microflora inhabiting the colon [86,90,91].

As previously stated, the initial phase of metabolism consists of the biotransformation of phenolic compounds via hydrolysis, oxidation and reduction. Because of the alterations in the phenolic compounds’ chemical structures brought about by these reactions, the compounds’ polarity is increased, which improves their solubility and facilitates their excretion. The majority of these reactions take place in the gastrointestinal tract, where the enzymes responsible for catalysing those reactions are either constitutively expressed by the tissues comprising the gastrointestinal tract (such as the CPY450 superfamily of enzymes) or secreted by the gut microbiota [90,91].

The hydrolysis of functional groups is facilitated by many different enzymes, the most significant of which are carbonic anhydrase, carboxylesterases and aldehyde dehydrogenase. Phenolic compound oxidation occurs through the action of the CPY450 superfamily. Finally, the majority of phenolic compound reduction reactions occur in the large intestine, where both enzymes secreted by the gut microbiota and native enzymes play a crucial role [92,93,94,95].

Commencing in the small intestine, the second phase of metabolism involves the integration of various chemical radicals into the phenolic compound’s chemical structure. Methylations and glucuronidations are the most common conjugation reactions. The second stage of metabolization continues after the absorption of phenolic compounds into the bloodstream, with most of them being metabolised in the hepatic tissues. The primary conjugation-based reaction observed in humans is glucuronidation, which consists in the binding of glucuronic acid to phenolic compounds, producing water-soluble compounds that are easily excreted. This reaction is catalysed by the enzyme uridine diphosphate (UDP)-glucuronide transferase. The methylation of phenolic compounds is less prevalent due to the fact that it typically reduces the solubility of those compounds in water and prevents the further metabolization of phenolic compounds. However, numerous phenolic compounds are methylated, with epicatechins and catechins being among the most prominent examples. The enzymes that catalyse these reactions are O-methyltransferases [96,97,98].

As a result of their extensive metabolism, virtually all phenolic compounds in circulation have undergone glucuronidation and/or sulphation. Furthermore, virtually no free aglycones can be found in plasma [99]. Flavonoids such as caffeic acid, quercetin and phloretin are notable exceptions. Plasma contains both conjugated and unconjugated forms of these compounds [100,101,102]. The detection of free flavonoid aglycones is also possible when these compounds are administered in a pharmacological dose. This suggests that at those concentrations, the conjugation pathways for these compounds can become saturated [77].

Even though the conjugation of phenolic compounds has been extensively studied and recognised, the majority of biological studies involving these compounds have exclusively utilised the free aglycone form. As a result, there is a limited understanding of the biological properties of conjugated derivatives [103].

Despite previous research demonstrating that sulphate esters and glucuronides retain a portion of their bioactivity, including antioxidant properties, recent studies have demonstrated that glucuronidation can significantly diminish the antioxidant and anti-inflammatory properties of flavonoids [104].

Additional research is required to comprehensively understand the potential applications of metabolites generated during phenolic compound metabolism (during which may retain a significant portion of their bioactivity), in addition to the impact of metabolism on the bioavailability and bioactivity of phenolic compounds [77,105]. The biological mechanisms by which phenolic compounds modulate food allergies are not yet fully understood, given that these compounds undergo extensive phase I and phase II metabolism and are poorly absorbed in the gastrointestinal tract. Therefore, further research employing phenolic compounds and their metabolites is required in order to comprehensively characterize the immunomodulatory properties of these substances.

## 4. Modulation of Food Allergies Using Phenolic Compounds

### 4.1. Modulation the Digestion of Food Allergens

The capacity of phenolic compounds to interact with proteins, both at the level of the food matrix and the human body, is one of their most crucial capabilities. This ability is at the origin of the different properties of these compounds, such as properties related to food science and technology (e.g., juices stability, taste properties) as well as being related to human bioactivities (e.g., digestive enzyme modulation) [51].

As discussed previously, protein stability is one of the defining factors of the immunogenic potential of dietary proteins. Proteins that can resist digestion in the gastrointestinal tract tend to preserve their structure and epitopes, therefore they tend to maintain their ability to initiate allergic reactions after being sampled by the cells of the immune system [21]. The ability of phenolic compounds to form both soluble and insoluble complexes with different proteins can reduce the allergenicity of dietary allergens, either by changing the structure of their epitope or by diminishing their bioaccessibility [106]. Dietary proteins, such as those found in seafood, milk and eggs, can form complexes through either irreversible covalent interactions or reversible non-covalent interactions. Both forms of interactions have the ability to modify the secondary and tertiary structures of proteins [107]. Immunogenicity can be altered by these modifications to protein structure, which may mask structural epitopes or alter their structure. Caffeic and chlorogenic acids, for instance, were reported to form complexes with milk proteins, thereby inhibiting their affinity for food-specific IgE [108,109]. Epigallocatechin gallate (EGGC) was shown to be able to covalently bind to egg allergens [110], while blueberry phenolic compounds were shown to non-covalently bind to peanut allergens [111]. In both cases, the secondary structure of these proteins was altered, thus altering their immunogenic potential. These modifications inhibit the binding of food-specific IgE. This could therefore reduce the allergenicity of these antigens and inhibit the degranulation of mast cells and basophils [107].

Alterations in protein structure may also impact the digestion process. An enhanced digestion of casein and whey proteins was observed as a consequence of the complexation with chlorogenic acid [112]. Nonetheless, phenolic compounds can also protect dietary protein from digestion, as is the case of wheat proteins treated with flavonoids extracted from onion skin [113]. This can prove a hindrance in the development of new therapeutic strategies, seeing as undigested proteins tend to keep their immunogenic potential [30]. Adding to this, an improved digestion of dietary proteins might not be enough to reduce their immunogenic potential, seeing as some large peptides formed during digestion maintain an ability to bind to IgEs [40]. One example of these are the milk proteins casein and α-lactoalbumin, which are vastly degraded by gastric pepsin in 2 min and yet retain their ability to initiate allergic responses [32].

Overall, the digestion of dietary proteins is a complex process, and many factors can contribute to the ability of different proteins to initiate allergic responses. Protein stability, preservation of epitopes, the relative abundance in foods and the effects of food matrixes in digestion are all important factors that determine the immunogenic potential of allergens [32,114]. The use of phenolic compounds to modulate the digestion of foods is an exciting new prospect but the full extent of this modulation is still not fully understood [115]. As seen in this section, different polyphenols interact differently with proteins, either protecting them from digestion or making them more susceptible to degradation [116]. Adding to this, many studies do not take in the account the effect of food matrixes in this modulation of the digestive process. More studies are then necessary to discern the full extent of the role of phenolic compound binding to dietary proteins in digestion, as well as its effect on the ability of proteins to bind to IgEs and initiate allergic responses.

### 4.2. Modulation of the Bioavailability of Food Allergens

The formation of complexes between phenolic compounds and dietary proteins can also enhance the bioavailability and preserve the bioactivity of phenolic compounds. The formation of these complexes prevents the metabolization of phenolic compounds in gastrointestinal tract, thereby facilitating their absorption and enabling them to exert their intended bioactivities further [117]. For example, authors have reported that protein-rich soybean flour protects anthocyanins from metabolism, thereby increasing their bioavailability [118]. Covertly, these phenolic compound–protein interactions might alter their hydrophilic characteristics and increase their molecular weight, altering their absorption and potentially reducing their bioavailability [119]. As such, more studies are needed to fully evaluate the effects of these interactions on the uptake of potentially immunogenic peptides.

Authors have also reported the ability of these compounds to bind to different families of receptors and transporters, altering their function and ability to translocate different molecules. It is thus possible that phenolic compounds can interact with membrane receptors in the intestinal epithelium, modulating the active transport of peptides and proteins across the intestinal barrier [119]. For example, the flavonoids quercetin, apigenin and kaempferol bind to OATP1A2 and OATP2B1, transporters which are localised in the apical membrane of the intestinal lumen [120]. The specific receptors involved in the sampling of dietary antigens by M cells and goblet cells are still not identified. Nonetheless, it is possible that dietary phenolic compounds could alter the sampling process, and thus more studies are required.

Finally, phenolic compounds have the ability to modulate the function and expression of tight junctions, as well as other proteins, possibly altering the influx of immunogenic proteins and peptides into the lymphatic tissues located underneath the intestinal lumen [121]. Plant phenolic compounds can regulate the NF-κB, MAPK, PI3K and PKC signalling pathways, reducing the localized inflammation of gut tissues and preserving the normal function and structure of tight junctions.

Overall, phenolic compounds can influence the intestinal barrier through a variety of complex mechanisms, preserving the normal intestinal uptake of potentially allergic peptides and proteins. However, some of these mechanisms and the possible interactions that exist between them are not fully understood. Adding to this, the number of in vivo studies focusing on this topic is limited, so the full implication of the modulatory effect of polyphenols in the bioavailability of allergens is yet to be fully understood.

### 4.3. Modulation of the Human Immune System

Phenolic compounds are capable of directly modulating the immune system. Several studies have demonstrated that these compounds possess the ability to regulate the immune response to food allergens through the inhibition of different enzymes involved in allergic reactions. For example, resveratrol has been shown to inhibit the cyclooxygenase family of enzymes in mice, thereby preventing the synthesis of prostaglandins. Prostaglandins are essential mediators of inflammation; therefore, inhibiting their synthesis could potentially mitigate localised inflammation in intestinal tissues [122].

Other phenolic compounds, like curcumin, inhibit IKK and MAPK in mice, downregulating the NF-κB signalling pathway and the MAPK signalling pathway, respectively [123]. As discussed previously, these signalling pathways are crucial for the degranulation of basophils and mast cells [50], and as such, their downregulation might prove useful for reducing the symptoms of food allergies.

Phenolic compounds can also inhibit the expression of specific enzymes that are implicated in the IgE-mediated allergic response to food allergens. The administration of epigallocatechin (EGCG) to human epithelial cells results in the inhibition of iNOS expression in macrophages, thereby leading to a decrease in the synthesis of critical inflammatory mediators [106].

Also susceptible to dietary phenolic compounds are the differentiation process and quantity of immune system cells. Male C3h/HeN mice that were treated with phenolic compounds extracted from fruit palm trees exhibited an increase in the number of Th1 cells in the intestinal lumen. Conversely, mice that were treated with the phenolic compounds baicalin and apigenin displayed a diminished count of Th2 cells. An increased count of Th2 cells is indicative of the deterioration of the oral tolerance to dietary antigens [124,125].

Phenolic compounds can also regulate the production of cytokines, either promoting a pro-inflammatory state through the production of IL-1β, IL-2, Il-6, IL-8 and TNF-α, or an anti-inflammatory state via the production of IL-10, IL-4 and TGF-β. Alterations to this equilibrium will have an impact on immune responses. It has been demonstrated that a number of phenolic compounds inhibit the expression of pro-inflammatory cytokines in various cell types, including activated human mast cell lines and lipopolysaccharide-activated mouse primary macrophages [126,127].

The major biological event associated with IgE-mediated food allergies is the degranulation of basophil and mast cells [50,128]. After the loss of oral tolerance, exposure to dietary allergens results in the production of food-specific IgE antibodies by immune system cells. Phenolic compounds have been used in the modulation of the degranulation of mast cells and basophils, even though the mechanism through which these modulations occur are still not completely understood. Nonetheless, some phenolic compounds reduce the secretion of pro-inflammatory mediators such as histamines and β-hexosaminidase. The degranulation of these cells is a hallmark of allergic reactions to orally ingested allergens and, as such, a decrease in the secretion of these pro-inflammatory molecules could reduce the symptoms associated with food allergies [129,130].

### 4.4. Modulation of the Human Oral Microbiota

As mentioned previously, the populations of commensal bacteria that reside in the oral cavity also greatly modulate oral tolerance to food allergens. The contributions of the oral microbiome to food allergies are often overlooked. Recent studies have highlighted the relation between several pathologies, either systemic or in the oral cavity, and dysbiosis of the oral microbiome. Changes in the oral microbiome can also alter the function of the host’s immune system [131].

The immunomodulatory role of the oral microbiome has been studied with animal models. The colonization of gastrointestinal tract of mice with *Klebsiella* bacteria isolated from the oral cavity of Crohn’s disease patients resulted in an inflammatory Th1 response, thus indicating the potential role of the oral microbiota as promoters of inflammation [132]. In another study, ligature-induced periodontitis resulted in the infiltration of B, Th17 and γδ Τ cells in the lamina propria of the intestines of mice, thus indicating that the oral microbiome and its composition could influence the populations of intestinal immune cells [133].

Human trial studies have also highlighted the importance of the oral microbiome in the maintenance of normal oral tolerance to food allergens [134,135]. In one of these studies, the microbiome of the oral cavity of patients with peanut allergies had a difference in composition and a lower phylogenetic diversity when compared with the oral microbiome of healthy individuals. Lower levels of *Bacteroidales*, *Bacillales*, *Lactobacillales* and *Streptophyta* were observed, while increased levels of *Neisseriales* were also reported. Along with these differences, the levels of oral SCFAs in individuals with peanut allergies were significantly lower than in healthy individuals. Finally, IL-4 secretion was increased in peanut-allergic subjects [136]. Studies like these point to a possible correlation between food allergies and dysbiosis in the oral microbiota.

The oral microbiome is the first one to be in contact with food and, therefore, the microorganisms rely on the compounds present in the ingested food. Diets high in dietary sugars reduce the populations of early colonizers like *Mitis streptococci*, allowing for the proliferation of potentially pathogenic microorganisms [137]. Their benefits rely on their increased capacity to adhere to teeth, their ability to grow more quickly and the synthesis of different compounds that inhibit the growth of cariogenic bacteria [137]. However, when this interspecies competition is disrupted, pathogenic processes take place. For example, the types of consumed foods can affect the pH level in the mouth, which in turn can influence the growth and survival of different oral bacteria. An excess of fermentable carbohydrates might upset the equilibrium between commensals and pathogens because the fermentation of carbohydrates can lead to an increase in the acidity of the mouth, promoting the growth of acidogenic bacteria, such as *Streptococcus mutans*. In contrast, a diet rich in fibre, whole grains, fruits and vegetables can promote a more alkaline pH in the mouth, which can support the growth of beneficial bacteria, such as Lactobacillus and Bifidobacterium [138].

While a large amount of research has been devoted to the effect of sugars in the oral microbiome, namely on pathogens, studies have only recently been focused on phenolic compound effects. Despite mounting evidence for phenolic compounds’ antimicrobial activity against some periodontal pathogens [138], other mechanisms through which they can modulate microbial populations, such as their anti-adherent ability and anti-inflammatory properties, need to be assessed to fully understand the interplay between phenolic compounds and the oral microbiome [139].

One of the most studied foods rich in phenolic compounds is green tea. It has been reported that tea consumption can consistently change oral bacteria in humans related to carcinogenesis [140]. A clinical trial investigated the effects of green tea phenolic compounds on the oral microbiome and immune-related parameters in patients with dental caries. The results showed that green tea phenolic compounds can modulate the oral microbiome by reducing the abundance of pathogenic bacteria and promoting the growth of beneficial bacteria. In addition, green tea phenolic compounds were found to reduce inflammation and improve immune function in patients with dental caries. Other phenolic compounds, such as the ones present in grape and red wine, exhibit a strong inhibition of the adherence of pathogenic microbiota to oral cells, thus preventing oral dysbiosis [141,142]. This protective effect could be useful in the management of not only periodontal diseases, but also food allergies.

In addition, oral microorganisms can metabolize those compounds [90]. Although these mechanisms are not as well explored as the metabolization of phenolic compounds by the gut microbiome, the metabolization of phenolic compounds starts in the oral cavity. Authors have described a moderate metabolization of glycosylated phenolic compounds by the glycosidases produced by the oral microbiome [143]. This will naturally affect their bioactivity and bioavailability, meaning that the immunomodulatory properties of phenolic compounds could also be altered.

Despite all of this, the role of the oral microbiome in the maintenance of oral tolerance and the progression of food allergies is still not as well understood as the role of the intestinal microbiome. Due to this, further research is needed to fully understand the mechanism through which this modulation occurs. Overall, the role of phenolic compounds in the human oral microbiome is complex and multifaceted, and more research is needed to fully understand their mechanisms of action. However, while the available evidence suggests that consuming foods and beverages rich in phenolic compounds can promote a healthy oral microbiome and reduce the risk of oral health problems, there is limited research on the specific effects of phenolic compounds on the oral microbiome in the context of food allergies.

### 4.5. Modulation of the Human Intestinal Microbiome

The human gut microbiome, is crucial in the normal function of many organs and biological processes. First, the human gut microbiome has a crucial role in the digestion of food [144], increasing nutrient harvest [145,146] and altering appetite signalling [147,148]. It also provides hosts with specific and unique enzymes and biochemical pathways. Many of the metabolic processes of the human microbiome are beneficial to the host, as they are involved in either the degradation of xenobiotics or nutrient acquisition [146].

The human gut microbiome acts as a physical barrier, protecting hosts against the excessive proliferation of potentially harmful pathogens through a combination of the secretion of antibacterial substances and competitive exclusion [59,149,150]. The maintenance of the normal intestinal microbiome is crucial for the maintenance of oral tolerance, as dysbiosis in the gut could lead to an abnormal function of the host’s immune system [151,152].

Research conducted using germ-free animals indicates that the microorganisms present in the gastrointestinal tract modulate the function of the immune system, being involved primarily in promoting the normal development of immune functions and the maturation of immune cells [152]. Germ-free animal models possessed abnormal levels of several immune cell types, had poor development of their GALT and thymus, smaller Peyer’s patches, mesenteric lymph nodes and differences in cytokine levels [151,153,154,155,156]. On the other hand, germ-free mice inoculated at birth with normal mouse intestinal microbiota no longer possessed an undeveloped immune system, thus reinforcing the importance of the interactions established between these microbial populations and their hosts [157].

Other studies have also called attention to the pivotal role of the gastrointestinal tract microbial population in the progression of many diseases, such as liver pathologies, metabolic disorders, infections, respiratory diseases and autoimmune diseases [64,158,159,160,161,162].

Experiments using germ-free animals have also confirmed the key role of intestinal microbiota in the regulation of oral tolerance. The exposure of germ-free animals to food allergens resulted in a loss of oral tolerance and subsequent exposures resulted in allergic reactions. However, the restoration of different microbial populations lead to a normal function of the immune system, with the establishment of Treg cell populations [163,164]. Cohort studies of patients with cow’s milk allergies have also revealed that a considerable intestinal microbial dysbiosis is common in these patients [165].

The gut microbiome and the oral microbiome, are not independent, as changes in one could alter the microbial ecosystem and bacterial metabolism in the other. Microorganisms primarily found in the oral cavity have been detected in the gastrointestinal tract of Crohn’s disease patients, colorectal cancer patients and HIV patients [132,150,166], while microbiota usually found in the oral cavity, like *Streptococcus*, *Pervotella*, *Rothia*, *Neisseria* and *Gemella* were detected in the stools of patients suffering from chronic intestinal inflammation [132]. Parallelly, inflammatory bowel disease (IBD) patients appeared to have a higher risk of developing pathologies in the oral cavity [167,168].

The composition and function of the gut microbiome can be modulated by phenolic compounds. Overall, research conducted in vitro and in vivo has demonstrated that phenolic compounds reduce the abundance of potentially harmful bacteria, such as *C. perfringens* and *C. histolyticum*, while increasing the quantity of advantageous *Clostridium*, *Bifidobacterium* and *Lactobacilli*. By resetting the dysbiosis that is characteristic of food allergy with phenolic compounds, abnormal immunogenic responses to dietary allergens can be regulated [169]. For example, authors have described that red wine phenolic compounds can act as prebiotics, promoting the maintenance of the normal gut microbiome by increasing the populations of the beneficial bacteria of the genus *Bifidobacteria*, *Bacteroides* and *Provotella* [170] and by decreasing the populations of bacteria typical of intestinal dysbiosis, *Escherichia coli* and *Enterobacter cloacae* [171]. In another study, proanthocyanin-rich extract from grape seeds also had the ability to positively modulate the composition of the human gut microbiome [172].

The modulation of the gut microbiome, using phenolic compounds could also result in changes in SCFA production. As explored in previous chapters, SCFAs are produced during microbial metabolism and have a plethora of immunomodulatory properties. Seeing as the production of these metabolites is dependent on the composition of the gut microbiome, the prebiotic effect of phenolic compounds could alter SCFAs production. Indeed, many authors have described increases in SCFA production in mice treated with different phenolic compounds extracts and isolated phenolic compounds [173,174,175,176,177].

The gut microbiome plays a role in the metabolization of orally ingested dietary phenolic compounds. These bacteria produce a vast array of enzymes with the ability to degrade phenolic compounds into new metabolites, with a different bioavailability and bioactivity [178,179]. This can in turn alter not only their ability to modulate the gut microbiome, and the metabolites it produces, but also their ability to bind to proteins in the small intestine and their ability to modulate the immune system cells. As such, the gut microbiome can significantly influence other biological systems [180].

## 5. Conclusions

The rapid increase in the prevalence of food allergies places an ever-increasing importance on the development of new strategies to not only manage symptoms, but also prevent these pathologies. Based on the mechanisms highlighted through this review, the use of foods rich in phenolic compounds could be the foundation for the development of new nutritional approaches, such as functional foods or supplements, that could decrease the severity of the symptoms associated with pathology, as well as improve patients’ quality of life.

Despite this, some missing links need to be addressed to successfully modulate immunological responses utilizing phenolic compounds. The bioactivity of both phenolic compounds and their metabolites must be carefully examined, as these substances undergo substantial metabolization after being consumed orally. Complex in vitro systems must thus be developed, enabling a deeper knowledge of the molecular and cellular mechanisms underlying this modulation, the impact of different bodily regions (such as the gut, mouth, blood, or blood vessels) in the metabolization of phenolic compounds and finally, the impact intervention window (prevention vs. treatment). Concerns about safety, such as the presence of harmful compounds from extraction processes, the cytotoxicity of the resulting extracts and the maximum advised daily intake, must also be addressed. The effectiveness and long-term safety profile of dietary phenolic compounds, whether used as therapy or dietary interventions, need to be assessed across a range of age groups. Therefore, more research is required to clarify existing mechanisms and possibly uncover new ones.

## Figures and Tables

**Table 1:** Immunomodulatory mechanisms already described by which phenolic compounds can be used as a powerful tool in controlling the prevalence of food allergies.

Phenolic Compound	Experimental Study	Biological Action	Food Allergy	References
Epigallocatechin gallate	Protein–phenolic compounds complexation	Conformational changes	Milk allergy (albumin)	[78]
Epigallocatechin gallate	Protein–phenolic compounds complexation	Conformational changes	Shrimp allergy (tropomyosin)	[79]
Resveratrol	Mouse model	Inhibition of Th2 differentiation and antigen presenting cells (APCs)	Ovalbumin	[80]
Red wine and coffee phenolic compounds	In vivo gut microbiota	Increase Bacteroides	Inflammation biomarkers of allergic rhinitis	[81]
Apple phenolic compounds extract	Mouse model	Reduction of allergy symptoms in a dose-dependent manner	Ovalbumin	[79]
Apple phenolic compounds extract	In vitro mast cell degranulation	Reduced histamine release	Universal allergy model	[82]
Phenolic acids	Protein–phenolic compounds complexation	Binding to peanut allergy-specific IgE	Peanut allergy	[83]

## Data Availability

Not applicable.
